# In Vitro Toxicological Insights from the Biomedical Applications of Iron Carbide Nanoparticles in Tumor Theranostics: A Systematic Review and Meta-Analysis

**DOI:** 10.3390/nano14090734

**Published:** 2024-04-23

**Authors:** Maria Antoniou, Georgia Melagraki, Iseult Lynch, Antreas Afantitis

**Affiliations:** 1 Department of Nanoinformatics, NovaMechanics Ltd., Nicosia 1046, Cyprus; antoniou@novamechanics.com; 2 Entelos Institute, Larnaca 6059, Cyprus; i.lynch@bham.ac.uk; 3 The Cyprus Institute, Nicosia 2121, Cyprus; 4 Division of Physical Sciences & Applications, Hellenic Military Academy, 16672 Vari, Greece; georgiamelagraki@gmail.com; 5 School of Geography, Earth and Environmental Sciences, University of Birmingham Edgbaston, Birmingham B15 2TT, UK

**Keywords:** iron carbide nanoparticles, nanomedicine, cytotoxicity, in vitro, metabolic activity, meta-analysis

## Abstract

(1) Background: Despite the encouraging indications regarding the suitability (biocompatibility) of iron carbide nanoparticles (ICNPs) in various biomedical applications, the published evidence of their biosafety is dispersed and relatively sparse. The present review synthesizes the existing nanotoxicological data from in vitro studies relevant to the diagnosis and treatment of cancer. (2) Methods: A systematic review was performed in electronic databases (PubMed, Scopus, and Wiley Online Library) on December 2023, searching for toxicity assessments of ICNPs of different sizes, coatings, and surface modifications investigated in immortalized human and murine cell lines. The risk of bias in the studies was assessed using the ToxRTool for in vitro studies. (3) Results: Among the selected studies (*n* = 22), cell viability emerged as the most frequently assessed cellular-level toxicity endpoint. The results of the meta-analysis showed that cell models treated with ICNPs had a reduced cell viability (SMD = −2.531; 95% CI: −2.959 to −2.109) compared to untreated samples. A subgroup analysis was performed due to the high magnitude of heterogeneity (I^2^ = 77.1%), revealing that ICNP concentration and conjugated ligands are the factors that largely influence toxicity (*p* < 0.001). (4) Conclusions: A dose-dependent cytotoxicity of ICNP exposure was observed, regardless of the health status of the cell, tested organism, and NP size. Inconsistent reporting of ICNP physicochemical properties was noted, which hinders comparability among the studies. A comprehensive exploration of the available in vivo studies is required in future research to assess the safety of ICNPs’ use in bioimaging and cancer treatment.

## 1. Introduction

In the last few decades, magnetic nanomaterials (NMs) have gained immense attention for applications in nanomedicine, owing to their distinct characteristics and unique ferromagnetic properties [1]. Magnetic nanostructures, such as iron-based nanoparticles (NPs), are widely reported in the literature as potential nanomedicine candidates [2,3]. They have shown promise in various biomedical applications, including targeted drug delivery [4], diagnostic imaging [5,6], tissue engineering, biosensing [3,7], and magnetic hyperthermia (MHT) [8]. Iron oxide nanoparticles (IONPs) are the most commonly employed NMs, among others [9], due to their straightforward synthesis process and relatively high biocompatibility [10,11]. However, it has been suggested that they may be unstable for extended use due to the release of iron ions [12,13], and their toxicity is still debatable [14]. Indeed, IONPs that were approved for clinical use have been withdrawn due to insufficient efficacy and/or a limited understanding of their biological impacts [15]. IONPs have, therefore, been characterized as unsuitable for applications in medicine [16], although the patent literature suggests that various formulations are still being developed for potential clinical use [17].

Recently, iron carbide (IC) nanostructures have been proposed as a candidate in nanomedicine, emerging as a promising material for cancer-related diagnosis and treatment [18,19]. ICNPs inherit the unique ferromagnetic and optical properties of iron, while their carbon content provides mechanical strength to the structure, as well as introducing strong covalent bonding to stabilize the NPs [20]. Since the carbon content contributes to the chemical inertness of the particle, and no iron ions are released, ICs are perceived as being a safer alternative to IONPs for health-related applications [18,20].

Several studies have proposed their use in bioimaging, serving as contrast agents in Magnetic Resonance Imaging (MRI) or Photoacoustic Tomography (PAT) [21,22,23]. MRI is a non-invasive clinical diagnosis modality extensively used in medicine for the provision of detailed anatomical information. Similar to other magnetic NPs, ICNPs possess superparamagnetic behavior, allowing them to enhance MRI contrast by affecting the relaxation times of surrounding water molecules [23]. In particular, Fe_5_C_2_ nanoparticles outperform traditional contrast agents like IONPs in generating hypo-intensities on T_2_-weighted MRI maps, leading to an improved contrast and visibility of the target against the background. This superiority is attributed to ICNPs’ significantly higher transverse relaxivity (r_2_) values compared to IONPs [19].

Aside from their possible use in diagnostics, they have been extensively studied for various tumor therapy applications [24,25]. Their high magnetization and heat-generating ability through magnetic relaxation render them suitable for Magnetic Hyperthermia. Further potential uses of ICNPs in tumor therapeutics include photothermal therapy (PTT) and photo- and chemo-dynamic therapy (PDT/CDT). Other applications involve enhanced drug release, where ICNPs’ magnetism and photothermal capacity enable controlled drug delivery [20]. Notably, however, there are limited patents relating to ICNPs for clinical applications in cancer as of yet [26].

Despite early indications of their successful implementation in bioimaging and therapeutics [19], a comprehensive review of the consequences of ICNP exposure to physiological environments is needed. To this end, a systematic review (SR) was conducted to collect and analyze data related to the material’s in vitro toxicity. This work uses the available scientific evidence published in the literature to search for studies that synthesized or utilized ICNPs and evaluated their cyto-toxicological effect in vitro, avoiding the traditional animal testing. Additionally, a meta-analysis quantitatively synthesized findings from multiple studies. An investigation of the material’s compatibility with physiological environments is essential for its safe incorporation in theranostics.

Similar systematic reviews have been conducted in the past, searching for the adverse effects of both organic and inorganic nanomaterials [27,28]. For instance, plenty of studies have provided insights into the potential risks associated with nano-titanium dioxide, a nanomaterial widely used in industrial products [29,30,31]. Other studies have explored the impact of silver [32,33] and silica NPs [34] in humans and animals. The findings of these studies included evidence of oxidative stress, genotoxicity and neurotoxicity, and inflammatory responses associated with NMs, observed in both in vivo and in vitro settings. In comparison to previous reviews, this SR concentrates on ICNPs and aims to summarize the existing evidence regarding their potential health impacts within biomedical applications.

## 2. Materials and Methods

### 2.1. Study Design and Protocol

**Figure 1:**
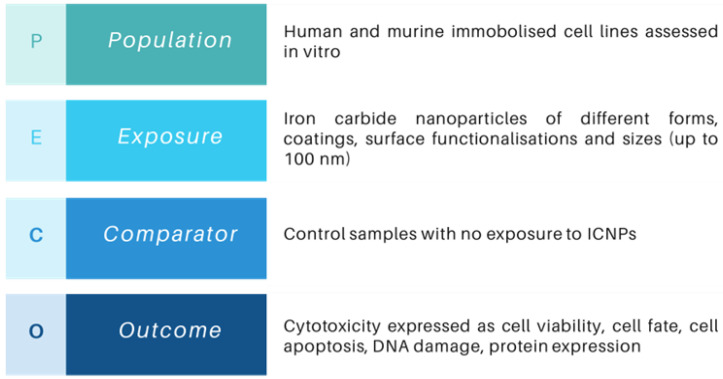
Description of the PECO statement for the SR which indicates the eligibility (inclusion/exclusion) criteria.

The objective of the present SR is to gain insights into the biocompatibility and health-related consequences of ICNPs applied in a diagnostic or therapeutic contexts. The Population, Exposure, Comparator, and Outcome (PECO) framework [35] was utilized to frame the research question of this SR and to assist in defining the eligibility criteria. The PECO strategy, as presented in Figure 1, formulated the review questions, such as: “Are iron carbide nanoparticles (ICNPs) suitable to use in various biomedical applications or is there a risk of adverse effects and toxicity in physiological environments? What nanotoxicological data related to ICNPs exist so far, relevant to the diagnosis and/or treatment of cancerous cells?”. Following the formulation of the review questions, the authors established an a priori protocol, as described by van den Akker et al. [36]. The protocol was pre-registered in the Open Science Framework (OSF) Repository (https://osf.io/84rv5, accessed on 26 March 2024) [37]. In addition, the SR is reported, where applicable, in accordance with the Preferred Reporting Items for Systematic Review and Meta-Analyses (PRISMA) guidelines [38,39]. Adherence to these guidelines ensures the transparency and reproducibility of the methodology followed in this work.

### 2.2. Eligibility Criteria

The inclusion and exclusion criteria were defined based on the PECO strategy, following a well-established approach for delineating the search results [40]. The “Population” component refers to the specific group being observed. To restrict the heterogeneity of the results, this study focused on the use of either healthy or immortalized (cancer) cell lines extracted from human and murine organisms, where the adverse effects were investigated in vitro. Cells from various organs or tissue sources, such as lungs, blood, ovaries, brain, and others, were eligible for inclusion. We also considered cells of different morphologies (epithelial or fibroblast) and included both adult and embryonic cells. The ‘Exposure’ element denotes the intervention applied to the cell lines, which, in this case, is exposure to ICNPs. Specifically, the interest of this study lies in the investigation of spherical nanoparticles whose size ranges from 1 to 100 nm, due to their unique properties, simplified structure, and wide use in the scientific literature [41]. Therefore, we considered studies which utilized NPs of different forms and phases (i.e., Fe_3_C, Fe_5_C_2_, and Fe_7_C_3_) and diverse surface modifications, such as core/shell structures or those conjugated with small molecules or ligands. Regarding the ‘Outcome’ aspect of PECO, the inclusion criteria specified studies that evaluated the biosafety of ICNPs using at least one endpoint at the cellular level. A variety of biochemical metrics, including cell metabolic activity, Reactive Oxygen Species (ROS) levels, lysosomal uptake, and cytokine and chemokine releases, were eligible for inclusion in this SR. 

After defining the above, the exclusion criteria were assembled to better refine the search results. The focus of this SR is to determine the consequences of ICNP exposure on a cellular level and gain insights into their possible toxicity without involving living organisms, i.e., based on in vitro studies only. Owing to this, studies that explored adverse effects in vivo were ineligible for inclusion. Additionally, in order to maintain consistency and allow for effective comparisons between the organisms of the study, ‘Population’, no cell lines other than human or murine were included. Since only spherical ICNPs were considered, other structures or shapes of NMs (i.e., carbon nanotubes, nanowires, nanorods, and nano-onions) were excluded from the search. Lastly, studies that evaluated the cytotoxicity of ICNPs using merely qualitative methods were also excluded to ensure reliable data extraction. 

### 2.3. Search Strategy

Upon the establishment of the eligibility criteria, a rigorous search of research articles relevant to the scope of this SR was performed. The search strategy involved querying three major databases, PubMed (Medline) [42], Scopus [43], and Wiley Online Library [44]. These databases, which are widely used for SRs [45], were selected because they are considered to offer a broad coverage of disciplines and their records are easy to retrieve. Their search systems also allow for different levels of recall and precision based on the specific requirements of the review. Gray literature, such as conference proceedings, pre-prints, theses, and reports, was not involved in the search strategy. Instead, only traditional peer-reviewed research articles indexed in the selected bibliographic resources were included. Web search engines (e.g., Google Scholar) were not explored, given that their functionality is crawler-based and they do not allow automatic record retrieval. Lastly, the strategy was limited to English-language studies that were published between January 2010 and December 2023. The search on the databases was completed in December 2023.

For optimization of the search strategy, three key concepts [46] were identified from the research questions: ICNPs, cytotoxicity, and biomedical applications. Then, the most crucial elements from each topic were determined. A balance between sensitivity and specificity was adopted when formulating the terms and elements included in the search query [47]. A high sensitivity in the search query would lead to a higher proportion of studies irrelevant to the scope of the SR and increase the screening time significantly, while a high precision would pose the risk of missing relevant research. In order to ensure sensitivity in the search terms, general concepts such as ‘cancer’ or ‘tumor’ were avoided and different forms/phases of ICNPs were included. On the other hand, to make the results more precise, specific terms were restricted to the title/abstract and included the terms ‘in vitro’ and ‘nanomedicine’. The main keywords and search terms used in the query were:

1. “Iron carbide” OR “Fe_x_C_y_” OR “Fe_2_C” OR “Fe_3_C” OR “Fe_5_C_2_” OR “Fe_7_C_3_”, AND
2. cytotoxicity OR toxic OR “cell viability” OR adverse OR “in vitro”, AND
3. biocompatibility OR biomedical OR nanomedicine OR theranostics OR mri OR “contrast agent” OR hyperthermia

Notably, the three selected databases have diverse functionalities, syntaxes, and field codes. For instance, PubMed employs Medical Subject Headings (MeSH) terms, while the remaining resources offer extensive thesauri [45]. Consequently, the search query was adapted according to the specific requirements and indexing methods of each interface, using Boolean operators, parentheses, quotation marks, and post-query filtering. The extended search queries, post-query refinements, and total numbers of results from each interface are shown in Table S1.

### 2.4. Screening and Selection Process

After the initial identification of the search results, peer-review citations were extracted from the major databases. The JabRef Bibliography Management software (version 5.12) was utilized for the management of the citations and the removal of non-English-language studies and gray literature. The process of removing duplicate records, as well as reviewing and managing the included articles, was streamlined with the Rayyan software (https://www.rayyan.ai/) [48]. There were three independent reviewers (M.A., G.M. and A.A.) involved in the selection process.

The screening procedure was divided into two separate stages. During the preliminary stage, two independent examiners (M.A. and G.M.) separately evaluated the collected papers’ titles, abstracts, and keywords, and determined which were suitable for inclusion and which were unrelated to the scope of this SR. Relevant review research articles were considered in the first stage of screening for possible citation searching, serving as valuable sources for articles unavailable in the three databases [49]. At the secondary stage of screening, the same examiners assessed the full-text manuscripts according to the aforementioned criteria for inclusion and exclusion, eventually detecting the eligible studies. Discrepancies were solved by the third examiner (A.A.), who supervised the review process and provided consultation throughout the two stages.

### 2.5. Data Extraction

**Figure 2:**
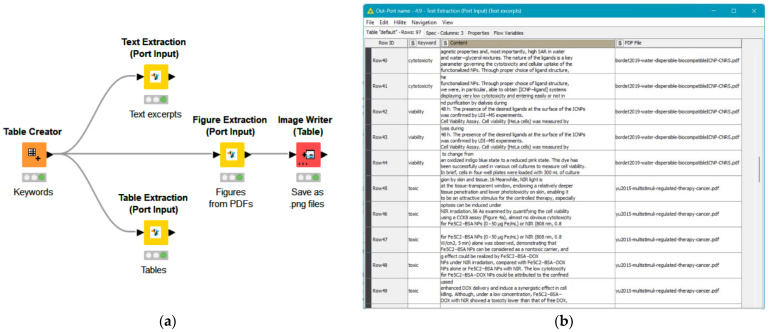
Text mining extension nodes in KNIME: (**a**) workflow of the Text, Figure, and Table extraction nodes connected with input keywords and (**b**) output of the ‘Text Extraction’ node with excerpts from the surrounding text of each located keyword in the inserted files.

Following the identification of appropriate studies, the data extraction process was carried out using the Enalos text mining KNIME extension nodes, as illustrated in Figure 2. The ‘Text Extraction’ node searches for specific keywords provided by the user and returns the surrounding text, while the ‘Table Extraction’ node extracts the relevant tables from the inserted files. The ‘Figure Extraction’ node was employed to access the graphs available in each manuscript, including the labels and the page numbers on which the figures in each file could be found. The employment of these nodes facilitated direct information retrieval, significantly reducing the data extraction time and automating the process through an easy-to-use interface. More information on the text mining nodes can be found at https://enalosnodes.novamechanics.com/textmining-nodes.html (accessed on 16 February 2024).

One reviewer (M.A.) utilized the KNIME workflow (Figure 2a) to obtain text passages, graphs, and tables from the publications included in the SR. Manual verification of the results was also performed to ensure the reliability of the automated procedure. The remaining reviewers provided consultation during the data extraction phase and assessed the recorded data. The extracted data were registered in a predesigned spreadsheet in tabular format for subsequent analysis. Where the desired data were given in graph plots, articles with low image resolution or graphs that were poorly labeled were excluded from the data extraction process. Otherwise, the PlotDigitizer (v. 2.6.9) freeware was utilized to extract the approximate average values, while standard deviations/standard error of measurement details were also gathered.

The parameters gathered from each study were the characteristics of the investigated ICNPs, such as the core/shell material and surface functionalization, their size and dose, and other physicochemical properties of the NPs, such as surface charge and magnetization saturation (Ms). The collected data were harmonized where necessary to ensure consistency in units. Moreover, the biochemical metric used to evaluate the biosafety of the material was recorded, including information regarding the immortalized cell model, cell source, exposure duration, and in vitro assay used to determine cytotoxicity. Where mentioned, the experimental conditions and culture medium (presence/absence of serum and % serum) were extracted. Any of the above that were consistently reported in all of the studies selected for inclusion were treated as primary outcomes, while columns in which some entries (ICNPs) were missing data were regarded as secondary outcomes and reported separately. 

### 2.6. Critical Appraisal

All the studies that were selected after the whole screening process underwent quality evaluation to ensure the reliability of their findings. The risk of bias was assessed using the Toxicological data Reliability Assessment Tool (ToxRTool, https://joint-research-centre.ec.europa.eu/scientific-tools-and-databases/toxrtool-toxicological-data-reliability-assessment-tool_en, accessed on 16 February 2024) [50] for in vivo and in vitro toxicity studies performed with NMs. This approach evaluates different criteria divided into five groups: substance identification, organism characterization, study design description, study result documentation, and plausibility of study design and results. The criteria in each group are represented by questions that need to be answered with ‘yes’ (1) if the study complies or ‘no’ (0) if it does not comply. Notably, the list contains ‘red’ questions, whose compliance is mandatory for a study to be rated as reliable. Following the principles of Klimisch scores [51], the studies are categorized as ‘reliable without restrictions’, ‘reliable with restrictions’, or ‘not reliable’, based on their overall score. 

### 2.7. Statistical Analysis

A meta-analysis was performed in order to derive more insights into the effects of ICNPs treatment on cell lines in vitro. The toxicity endpoints referred to continuous outcomes, thus, the effect size (ES) between the samples treated with NPs and the control groups was defined as the standardized mean difference (SMD) and the corresponding 95% confidence interval (CI), using the Hedges’ g method for ES estimation [52]. A random-effects model was considered to analyze the pooled ESs due to the expected heterogeneity among the included studies. The assumption underlying the random-effects model is that intervention effects are normally distributed and consider between-study variability [53]. A significance level of *p* < 0.05 was adopted as a statistical threshold and the inverse variance method was chosen to assign weights for each experiment. The effect estimates were illustrated using forest plots. 

Statistical heterogeneity among studies was determined with Cochran’s Q statistic, where *p* < 0.05 represented heterogeneity. The I^2^ index was also used, whose values of <50%, 50–75%, and >75% were, respectively, considered for low, moderate, and substantial heterogeneity [52]. Publication bias was examined with funnel plots and Egger’s linear regression analysis [54,55]. No publication bias is suggested if the funnel plot is symmetrical and Egger’s *p*-value surpasses 0.05, otherwise, the trim-and-fill method [56], a delicate statistical approach which involves non-trivial computing procedures, is needed. Furthermore, subgroup analyses were implemented to identify potential sources of heterogeneity among studies. Treatment variables such as the NP size of the ICNPs, treated organism, cell line health status, conjugated ligands, and NP concentration were analyzed to examine their association with potential cytotoxicity. All statistical analyses were performed with R software (version 4.3.2), using the ‘meta’ package (version 7.0-0).

## 3. Results

### 3.1. Literature Search

**Figure 3:**
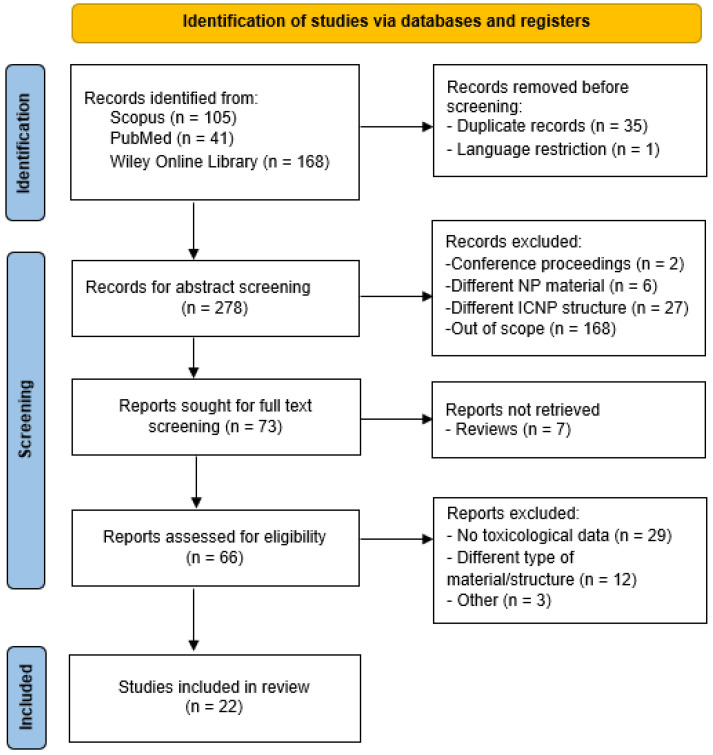
PRISMA 2020 flow diagram of the eligibility assessment process applied in the current SR.

This search resulted in an initial selection of 314 peer-reviewed original research articles (105 from Scopus, 41 from PubMed, and 168 from Wiley Online Library) reporting on the synthesis or biomedical application of ICNPs, where a cytotoxicity evaluation was performed using in vitro cell systems. The PRISMA flowchart for databases and registers, depicted in Figure 3, demonstrates the selection process of relevant publications at each stage of the SR workflow. Following the removal of 35 duplicate records and 1 publication in German, a total of 278 different research articles remained after Stage 1. 

After all titles, abstracts, and keywords were screened, articles reporting in the wrong publication type, reporting on the wrong population or exposure type, and completely irrelevant studies were removed. During the second screening stage, review articles were removed from the publication pool. No additional peer-reviewed articles were identified through the ascendancy approach. Subsequently, 66 articles were deemed eligible for full-text evaluation. Although many studies reported the synthesis of ICNPs and suggested their application in cancer-related theranostics, only twenty-two actually performed toxicity assessments and were selected for inclusion in the SR. Further details on the reasons for the exclusion of post full-text screened publications are available in Table S2.

### 3.2. Characteristics of the Included Studies

The foremost aim of this SR was to quantify the cytotoxic effects on healthy and cancer cells caused by exposure to ICNPs. Therefore, the initial priority after collating and the appropriate studies was to determine the biochemical metrics used for toxicity assessment. Identifying the primary toxicity endpoint most consistently used in these articles restrained the diversity in study designs and facilitated comparability between the selected studies. Specifically, a variety of biomarkers were employed to express the biological response from the cells, namely, the cell metabolic activity, cellular internalization, lactate dehydrogenase (LDH) release, cytokine profiling, live/dead assays, and ROS detection. The reviewed studies are summarized in Table 1, clustered according to the metrics for evaluating cytotoxicity.

Cellular metabolic activity prevailed as the most commonly measured toxicity endpoint, with 20 out of 22 studies reporting on it. Precisely, the studies presented the cell survival percentages after exposure to ICNPs of different concentrations, using suitable assays and indicators. However, quantitative results were obtainable from only sixteen of these studies, due to inadequately labeled graphs or images of a low resolution. Cell uptake assays, tracking the intracellular fate of NPs within a cell, as well as live/dead assays using fluorescent straining to assess the viability of cells after ICΝP exposure, were commonly used across the studies. Despite their effectiveness in cyto-toxicological evaluation, these assays provide merely qualitative observations in most cases, thus, the extraction of quantitative data was hindered.

**Figure 4:**
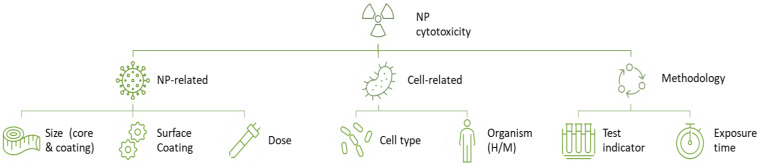
Summary of key features extracted from the included studies of ICNP in vitro cytotoxicity.

Relevant data were extracted from each study, as described in Section 2.5. Eventually, there were 14 different studies in the meta-analysis from 16 research articles, as presented in Table 1. The primary elements that were consistently reported and carefully extracted from the included literature are illustrated in Figure 4. Features describing the NPs included the core and shell materials, surface modifications of the ICNPs, and the ICNP concentrations applied to cells. Information relevant to the cell models used for the assessment, such as the cell type and whether they were human or murine cells, were also extracted. In addition, attributes regarding the methodology used for toxicity assessment, namely, cytotoxicity assay and incubation time, were methodically extracted. Table 2 summarizes the effects of ICNP exposure on cell viability, organized by concentration range. 

In contrast, physicochemical properties describing the ICNP surface charge, magnetic characteristics, and additional experimental conditions were not consistently investigated/reported across all the selected studies. The majority of the studies lacked measurements of zeta potential (ZP), a marker commonly used to approximate the surface charge of NPs. These properties were treated as missing data (Table S3). Where the ICNPs were investigated as contrast agents in MRI, magnetization saturation was often measured to interpret the NPs’ interactions with magnetic fields. Considering that ICNPs are also proposed as potential photothermal agents, certain studies treated the NPs with near-infrared (NIR) laser irradiation [61,66,78]. These studies evaluated the alterations in cell toxicity post-treatment, thus, NIR treatment was added as a binary attribute (yes/no) to the collected data (Table 2).

Comprehensive information on the immortalized cell models employed across the selected studies is presented in Table 3. Cytotoxicity assays of ICNPs were performed on human and murine cell lines of diverse ages and morphologies among the studies. The exposed human cells included mostly malignant types, such as the lung adenocarcinoma cell line A549, breast cancer MCF-7, MDA-MB-231 and SK-BR-3, cervical cancer HeLa, ovarian carcinoma SK-OV-3, and brain glioblastoma U87MG cell lines. On the other hand, cell lines isolated from murine organisms were mainly normal types, such as the mouse fibroblasts NIH-3T3 and L929 and monocyte/macrophage-like cell line RAW264.7. The greater part of the selected studies utilized the HeLa cell model, while many studies incorporated cells from both organisms (human and murine) for the toxicity assessment. 

### 3.3. Quality Assessment 

Many of the studies did not provide relevant information about including a positive control. Although the inclusion of a positive control is marked as an essential criterion in the ToxRTool, studies without a positive control were not categorized as ‘Not reliable’, as suggested by Schneider and colleagues [50]. Instead, this criterion was combined with the one for negative control, and its compliance was regarded as optional. However, studies without control groups, where cell lines were not treated with ICNPs (i.e., did not have an untreated control), along with studies that did not disclose the number of experiment replications, were excluded from the meta-analysis. The assigned categories of each selected study are presented in Table 4. Since all studies complied with the essential (“red”) criteria, none were classified as ‘not reliable’. Out of the 14 studies assessed, 12 were assigned 14–17 points and classified as ‘reliable without restrictions’ and 2 studies were graded as ‘reliable with restrictions’.

### 3.4. Meta-Analysis 

Upon reviewing the remaining experiments, after the removal of studies lacking an untreated cellular control, it was observed that the NIR-treated samples’ cell viability was significantly decreased and were, therefore, analyzed separately. This resulted in k = 172 cell viability data samples from 11 studies being included in the meta-analysis. 

**Figure 5:**
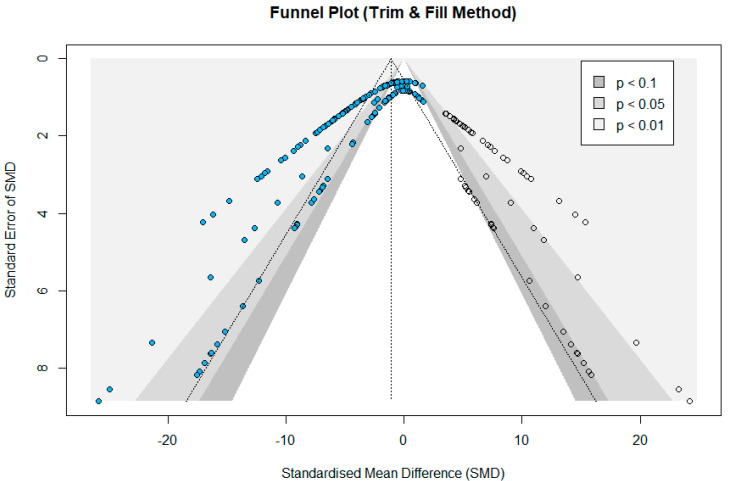
Funnel plot for assessing publication bias of the included studies describing the toxicity of ICNPs to cell lines that were included in the meta-analysis. Blue dots represent the original samples, and hollow dots represent the samples added with the trim-and-fill method.

Overall, the results showed that ICNP treatment had a significant effect on the immortalized cell lines’ survival compared to the control group, with an SMD of −2.531 (95% CI: −2.959 to −2.102) under a random-effects model. An SMD = 0 indicates no difference between the experimental and control groups, whereas an SMD < 0 suggests a higher cytotoxicity over the exposure group compared to control conditions. A high heterogeneity was observed between the included studies, with an I^2^ index of 77.1% and Q(171) = 745.90 (*p* < 0.001). The forest plots, illustrating the individual experiments’ effect sizes and their corresponding CIs, are provided in the Supporting Material. The presence of publication bias was observed by the non-symmetrical funnel plot that was initially generated and the result of Egger’s test, which was *p* < 0.05. Specifically, a higher proportion of the points was positioned on the left side of the funnel plot’s vertical line that represents the combined effect value (SMD), reflecting an uneven distribution. Thus, the trim-and-fill method was used in order to eliminate publication bias. As depicted in Figure 5, an additional 57 points were added, indicating studies that needed supplementation, to balance the inclination towards the INCP-treated group.

The factors contributing to the high variability in Ess across studies were interpreted using subgroup analyses (Figures S1–S3). On the basis of the species of the cells, the results indicated that, compared to the reference group, those isolated from humans (SMD = −2.791; 95% CI: −3.383 to −2.199) had a greater reduction in cell viability than those derived from murine organs or tissues (SMD = −2.288; 95% CI: −2.849 to −1.225). The influence of the cell lines’ health status was explored, confirming more pronounced toxic effects in cancer cell lines (SMD = −2.711; 95% CI: −3.214 to −2.208) compared to the effects on normal (healthy) cells (SMD = −2.037; 95% CI: −2.849 to −1.125). In the subgroup analysis of particle size, NPs were divided into smaller than 20 nm (SMD = −2.761; 95% CI: −3.577 to 1.947) and larger than 20 nm (SMD = −2.456; 95% CI: −2.965 to −1.947), and both had a negative impact on the outcome measure.

The subgroup analysis of the different ligands used to stabilize the ICNPs revealed that PEG-based ligands (SMD = −4.062; 95% CI: −4.835 to −3.289) had a large effect on the survival of cells. A mild influence was observed when protein-based ligands (SMD = −2.280; 95% CI: −3.092 to −1.469) were used, while the toxic impact was relatively limited for NPs without conjugated ligands (SMD = −0.594; 95% CI: −0.997 to −0.191). Concentrations less than 10 μg/mL IONPs (SMD = −0.651; 95% CI: −1.209 to −0.093) showed minimal cytotoxic effects compared to the control groups, whereas in dose ranges of 10–50 μg/mL (SMD = −1.987; 95% CI: −2.713 to −1.261) and 50–100 μg/mL (SMD = −1.185; 95% CI: −1.589 to −0.781), a moderate decrease in cell survival was observed. The number of living cells was affected significantly when the ICNP concentration increased to 100–250 μg/mL (SMD = −3.358; 95% CI: −4.166 to −2.551) and above 250 μg/mL (SMD = −5.337; 95% CI: −6.768 to −3.906). Statistically significant differences (*p* < 0.001) were observed among different ICNP ligand types and concentration ranges, indicating variations in cytotoxic effects. Detailed information on the subgroup analysis is available in Table 5.

**Figure 6:**
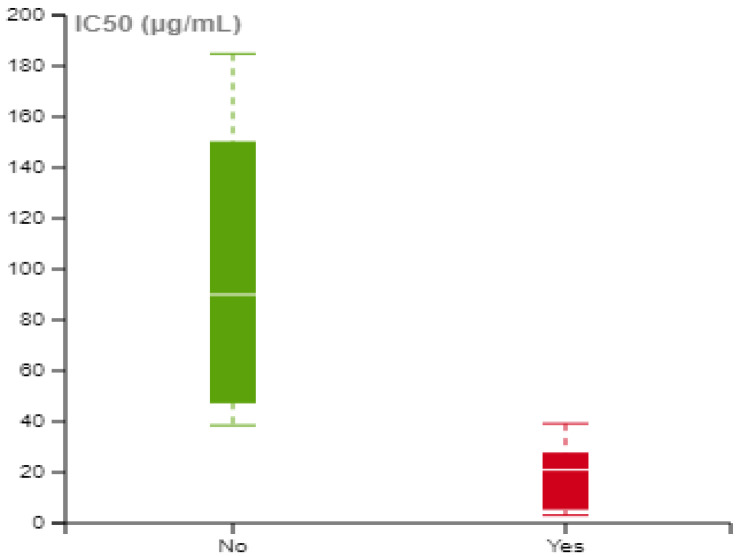
Half-maximal concentration at which 50% of the cells are dead (IC_50_) with and without NIR irradiation treatment.

To investigate the impact of NIR treatment on biological response, a focused analysis was conducted. Given that the extracted data were presented in a dose-dependent manner, it was vital to represent cell viability as a single point for each experiment, allowing for useful comparisons between different studies and experimental conditions. The half-maximal inhibitory concentration (IC_50_) was determined where appropriate, using the extracted data. This metric corresponds to the concentration at which the cell viability is reduced by 50% compared to the untreated control cells. For the calculation of IC_50_, the Enalos IC50 tool was employed, which uses a four-parameter logistic regression model that describes the response pattern with a sigmoid function [79]. However, calculations were not feasible in all the identified experiments, since, in many cases, cell viability levels exceeded 50% for the range of tested concentrations (Table S3). Figure 6 illustrates the IC_50_ values with and without NIR treatment. When irradiation was applied to the ICNPs, lower IC_50_ values were obtained, suggesting that NIR treatment enhanced the cytotoxic effects of the ICNPs on the cells.

## 4. Discussion

In the present systematic review, we evaluated the published evidence on the biosafety of iron carbide nanoparticles (ICNPs) in biomedical research, focusing on in vitro toxicity assessments. As stated in the ‘Results’ section, just 22 research articles out of a total of 314 papers initially identified were selected for this SR. The low percentage of papers complying with the eligibility criteria is attributed to the inclusion of the “toxic” and “in vitro” search terms, which dramatically increased the produced results without necessarily increasing the number of eligible papers. Several papers contained these search terms in their introduction or discussion without containing a biocompatibility assessment of ICNPs. On the other hand, removing this specific search term could limit the number of identified papers with a risk of losing articles of interest. 

### 4.1. Primary Outcomes 

A decrease in cell viability was the most frequently measured endpoint among the selected studies, using different cytotoxicity assays. Different cytotoxicity assessment methods were employed for the biosafety evaluation of ICNPs, with all included publications utilizing colorimetric indicator assays. In cellular metabolic activity assays, a decrease in the number of living cells results in a decrease in the metabolic activity of the tested sample. The majority of the included studies used MTT, a methyl-tetrazolium cytotoxicity assay. A few studies [58,75] employed sulforhodamine B (SRB), a protein-based colorimetric assay, while one study [66] used the CCK8 assay to assess the NPs’ toxicity. According to Keepers et al., the MTT and SRB cytotoxicity assays yield comparable results when assessing sensitivity in human tumor cell lines [80]. 

Overall, the majority of the selected publications reported a decrease in cell viability, both in murine and human cell models, following their exposure to ICNPs. Data synthesis revealed that the cell survival percentile continuously declined with increasingly high test concentrations, underscoring a dose-dependent relationship between ICNP exposure and cellular toxicity. The concentration-related adverse response of nanoparticles to biological systems has been extensively documented in the literature [81,82,83]. Notably, several studies [30,58,63,64] did not observe substantial decreases in cell viability following ICNP exposure. However, they employed relatively low maximum tested concentrations compared to the rest of the studies or were tested for shorter exposure durations (4 h), as compared to the 24 h incubation times used in other experiments. According to ISO standards for the performance of in vitro assays [84], a decrease in cell viability to less than 70% of the control sample is indicative of cytotoxic potential. Specifically, out of the k = 172 data points collected for the meta-analysis, just 25 of them fell below this threshold, suggesting a cytotoxic effect. These results provide insights into the potential health risks associated with ICNPs. An additional investigation of ICNPs’ mechanism of action and in vivo response is required to assess their safety profiles in theranostics contexts.

It is noteworthy that the majority of the selected studies lacked appropriate reporting of surface chemistry. Only a few of the studies measured the magnetization saturation after surface modifications. Several studies included cell viability measurements after NIR treatment. Zeta potential is a crucial NP physicochemical property that needs to be experimentally measured under specific exposure conditions, since it is usually utilized as a marker to approximate NPs’ surface charge. However, it is an extrinsic property, meaning that it is affected by the surrounding mediums’ ionic strength and pH. The charge of the functional group on the surface characterizes the interactions between the ICNPs and the cell membranes/tissues and affects the composition of the protein corona acquired by the NPs from the cell culture medium serum [85], consequently influencing the subsequent cellular uptake and cytotoxicity. Corona formation affects NPs’ interaction with cell membrane/cellular receptors and will be affected by the source, amount, and nature of the serum added to the system [86].

### 4.2. Meta-Analysis Outcomes

A meta-analysis was conducted using 11 of the selected publications. The results of the meta-analysis revealed that, collectively, ICNPs induce cytotoxic effects in human and murine cell lines tested in vitro. A subgroup analysis was performed due to the high magnitude of heterogeneity, which indicated that the toxicity of ICNPs is influenced by a variety of factors, such as the particle size, cell species, and health status of the cell. Additionally, the subgroup analysis revealed that the type of conjugated ligand and ICNP concentration range are statistically significant factors influencing cytotoxicity. 

Various stabilizing ligands were tested by the selected studies in an attempt to improve the biocompatibility of the ICNPs [87]. Notable differences were observed in the cytotoxicity of the ICNPs, depending on the type of ligands used for stabilization. For instance, studies employing ligands such as poly(acrylic) acid, protocatechuic acid, and Pluronic F127 acid exhibited lower degrees of cytotoxicity compared to those with polyethylene glycol (PEG) ligands. Although PEG-based ligands are among the preferred choices for NP stabilization [87], ICNPs conjugated with PEG ligands exhibited the most significant decreases in cell viability. The studies that utilized protein-based ligands showed a relatively milder impact on cellular response. Metallic NPs loaded with anti-cancer drugs, such as doxorubicin (DOX), showed more cytotoxic effects compared to DOX-free NPs [88,89]. Interestingly, bare ICNPs consisting of core/shell structures, devoid of conjugated ligands, displayed relatively limited toxicity. The varying cytotoxic effects of ICNPs observed after surface functionalization highlights the importance of considering the type of ligands in NP design for biomedical applications.

Furthermore, a dose-dependent relationship between ICNP concentration and cytotoxicity was observed, underscoring the importance of considering NP dosages to mitigate adverse effects on cell viability. Lastly, it was noted that NIR treatment is a factor that may potentially impact ICNP toxicity, which needs further investigation.

### 4.3. Strengths and Limitations 

The strengths of this SR and meta-analysis lie in its adherence to the PRISMA guidelines and recommended practices for SRs (Table S5) [90], assuring its transparent search and selection procedures, exhaustive data extraction, and rigorous data synthesis, and in its adherence to the Cochrane guidelines for meta-analysis. Multiple study designs are integrated in this analysis, extending the number of eligible publications relevant to the topic and assessing various endpoints for NM toxicity. 

There are also weaknesses in the present study. Limitations are introduced from the review process, since a single reviewer assessed the risk of bias and conducted the data extraction, while two other review authors inspected the processes and provided consultation. Furthermore, the eligible publications were limited to English-language and peer-reviewed articles. The restriction on gray literature could be a potential source of publication bias, as non-peer-reviewed sources, especially patent literature, can provide data not found in conventional database searches [91].

Moreover, a limitation of the included evidence is the inconsistency in the measurements and reporting of physicochemical properties such as zeta potential and magnetization saturation. These properties significantly affect the behavior of NPs and their interactions with cells. No authors were contacted in the case of missing data. Additionally, concentration rates and exposure durations varied between studies, potentially impacting the reliability of the data. The cytotoxicity of NPs, in general, is heavily dependent on their administered dose [83]; therefore, authors might have tested lower concentrations to prevent unwanted results, since ICNPs are intended for clinical use at sub-toxic levels. Lower incubation times might not allow adequate time for interactions between the NPs and the cellular models, thereby biasing the reported results. The data synthesis relied on the in vitro assessment of metabolic activity, omitting other biometrics such as cell death and protein expression. Lastly, in vivo studies were omitted, which, in future analyses, may capture valuable information on ICNP cytotoxicity and systemic toxicity.

## 5. Conclusions

In conclusion, a systematic review and meta-analysis were conducted to provide insights into the in vitro biocompatibility profiles of iron carbide nanoparticles (ICNPs), a promising nanomaterial for tumor theranostics. Peer-reviewed publications from January 2010 to December 2023 were systematically gathered, focusing on extracting information regarding exposure to ICNPs in biomedical contexts and their safety in physiological environments. This endeavor resulted in a robust dataset derived from 14 studies described in 16 publications, identifying the characteristics of the ICNPs with a diverse range of surface functionalization and conjugated ligands, the cell models to which the ICNPs were presented, the experimental conditions utilized, and the toxicity assessment methodologies and outcomes. The main findings of the meta-analysis included the substantial heterogeneity among the included studies, the dose-dependent effect of ICNP exposure on the reduction in cell survival, and the effect of biocompatible ligands conjugated onto the ICNPs. The unfavorable influence of NIR irradiation on cell viability was also unveiled. Although the outcomes of this review are encouraging for the potential application of ICNPs in biomedical research, further investigation is needed before their widespread use in clinical settings. The effect of their physicochemical properties should be explored, and continued investigation of the available in vivo studies and their toxicity mechanism is required. A comparison with the more widely studied iron oxide nanoparticles (IONP) may also be useful for broader contexts and potential read-across from this relatively data rich material.

## Figures and Tables

**Table 1:** Overview of the toxicity and biochemical metrics used in the reviewed studies. References are presented in chronological order.

a/a	Reference	Metabolic Activity	Cell Uptake	LDH Release	Cytokine Analysis	Fluorescent Live/Dead	ROS Detection	Other
1	Herrmann, 2011 [57]		√ *	√	√			
2	Sharma, 2012 [58]	√						
3	Schumacher, 2013 [59]			√	√			
4	Tang, 2013 [60]	√	√ *			√ *		
5	Yu, 2014 [61]	√				√ *		
6	Izydorzak-Wozniak, 2014 [62]	√ **						√
7	Huang, 2014 [63]	√						
8	Cowger, 2015 [64]	√	√					
9	Jacobson, 2015 [65]	√ **	√	√ **				
10	Yu, 2015 [66]	√						
11	Herrmann, 2016 [67]	√ ***						
12	Hasan, 2017a [68]	√						
13	Hasan, 2017b [69]	√ ***						
14	Feng, 2017 [70]	√	√ *			√ *	√	
15	Ahmadpoor, 2018 [71]	√						
16	Feng, 2018 [72]	√	√ *			√ *	√	
17	Bordet, 2019 [73]	√	√					
18	Yu, 2019 [74]	√	√ *					
19	Gangwar, 2020 [75]	√				√ *		
20	Sun, 2021 [76]	√					√	
21	Zhao, 2021 [77]	√	√ *			√ ***	√	
22	Ülküseven, 2023 [78]	√				√		

* No quantitative data; ** Test were performed but no results were given; *** Graph inadequately labeled, Image of low resolution.

**Table 2:** Characteristics of the ICNPs and their effects on cell viability by concentration range obtained from the studies included in the meta-analysis ^a^.

Ref.	Iron Carbide	Shell	Ligand- Conjugate	Size (nm)	Exposure (h)	Organism	NIR	Dose Range (μg/mL)	Cell Viability Range (%)
[58]	Fe_3_C	Carbon	-	92	24	H/M	No	0–500	100–81.7% (H), 100–81.9% (M)
[60]	Fe_5_C_2_	Fe_3_O_4_	DSPE-PEG-COOH	22	24	H	No	0–25	102–80.5%
[61]	Fe_5_C_2_	Carbon	Zher2:342	20	24	H/M	No	1–1000	105–91.8% (H), 103–91% (M)
	Fe_5_C_2_	Carbon	Zher2:342	20	24	H	Yes	1–1000	70–19.5%
	Fe_5_C_2_	Carbon	PEG	20	24	H	Yes	1–1000	102–44.6%
[63]	Fe_5_C_2_	Carbon	ST	20	24	H	No	0–100	100–82.6%
[64]	Fe_5_C_2_	-	DSPE-PEG-COOH (PL)	5	4	M	No	0–100	100–84.9%
	Fe_5_C_2_	-	DSPE-PEG-COOH (PL)	14	4	M	No	0–100	100–82.1%
	Fe_5_C_2_	-	DSPE-PEG-COOH (PL)	22	4	M	No	0–100	100–86%
	Fe_5_C_2_	-	ZDS	22	4	M	No	0–100	100–87.2%
	Fe_5_C_2_	-	Casein	22	4	M	No	0–100	100–88.5%
[66,70]	Fe_5_C_2_	Carbon	BSA-DOX	20	24	H/M	No	0.05–50	100–71.5% (H), 102–63.8% (M)
	Fe_5_C_2_	Carbon	BSA-DOX	20	24	H	Yes	0.05–50	95.9–5%
	Fe_5_C_2_	Carbon	BSA	20	24	H	No	0.05–50	101–91.7%
	Fe_5_C_2_	Carbon	BSA	20	24	H	Yes	0.05–50	97.7–41.5%
[68]	Fe_2_C	-	PA	16	24	H	No	0–400	92.1–91.3%
	Fe_2_C	-	-	16	24	H	No	0–400	91.7–16.2%
[71]	Fe_5_C_2_	SiO_2_	-	58	24	H	No	0–500	100–35.2%
[72]	Fe_5_C_2_	MnO_2_	GOD	22.1	24	H	No	0–800	100–26%
	Fe_5_C_2_	MnO_2_	-	22.1	24	H	No	0–800	101–98%
[73]	Fe_2.2_C	-	DOP-TEG-C6	15	4	H	No	10–2000	95.3–1.7%
	Fe_2.2_C	-	DOP-TEG-C6	15	24	H	No	10–500	64.8–2
	Fe_2.2_C	-	DOP-TEG-COOH	15	4	H	No	10–2000	104.5–85.5%
	Fe_2.2_C	-	DOP-TEG-COOH	15	24	H	No	10–500	105.4–59.4%
	Fe_2.2_C	-	DOP-TEG-Zwitter	15	4	H	No	10–2000	95.4–93.2%
	Fe_2.2_C	-	DOP-TEG-Zwitter	15	24	H	No	10–500	95.4–62.3%
[74]	Fe_5_C_2_	Fe_3_O_4_	DSPE-PEG	20	24	H/M	No	0–400	100–72.6% (H), 100–56.8% (M)
	Fe_5_C_2_	Carbon	DSPE-PEG	20	24	M	No	0–400	100–78.1%
[75]	Fe_3_C	-	Pluronic acid F127	6	24	H	No	0–300	100–76%
	Fe_3_C	-	Pluronic acid F127	6	48	H	No	0–300	100–73.1%
	Fe_3_C	-	-	6	48	H	No	0–300	100–73.7%
[76,77]	Fe_2_C	Fe_3_O_4_	DSPE-PEG	14	24	H/M	No	0–400	100–59.9% (H), 100–62.8% (M)
[78]	Fe_3_C	Carbon	PAA	45	24	H/M	No	0–200	100–73.7% (H), 100–87% (M)
	Fe_3_C	Carbon	PAA	45	48	H/M	No	0–200	100–47% (H), 100–84.4% (M)
	Fe_3_C	Carbon	PAA	45	24	H	Yes	0–200	100–6.4%

^a^ Abbreviations: DSPE-PEG, 1,2-Distearoyl-sn-glycero-3-phosphoethanolamin—Polyethylene Glycol; ST, Sodium Tartrate; PL, Phospholipids; ZDS, Zwitterion Dopamine Sulfonate; BSA, Bovine Serum Albumin; DOX, Doxorubicin; PA, Protocatechuic Acid; GOD, Glucose Oxidase; DOP, Dopamine; TEG, Triethylene Glycol; PAA, Poly(acrylic acid); H, Human; M, Murine.

**Table 3:** Information on the immortalized cell lines utilized in the studies included in the meta-analysis ^a^.

Organism	Cell Line	Source Organ/Tissue	Cell Type	Health Status	Age	Utilized in Studies
Human	A549	Lung	Epithelial	C	A	[75]
	HEK-293T	Kidney	Epithelial	N	E	[68,74]
	HeLa	Cervix	Epithelial	C	A	[58,61,63,68,72,73,74,77,78]
	MCF-7	Breast	Epithelial	C	A	[58,71,78]
	MDA-MB-231	Breast	Epithelial	C	A	[77]
	SK-BR-3	Breast	Epithelial	C	A	[61]
	SK-OV-3	Ovaries	Epithelial	C	A	[61,66]
	U87MG	Brain	Glioblastoma	C	A	[60]
Murine	4T1	Mammary Glands	Epithelial	C	A	[74,77]
	L929	Adipose	Fibroblast	N	E	[58,74,78]
	NIH-3T3	Embryos	Fibroblast	N	E	[61,66]
	RAW 264.7	Blood	Macrophage	N	A	[61,64,66]

^a^ Abbreviations: C, Cancer cells; N, Normal cells; A, Adult cells; E, Embryonic cells.

**Table 4:** Information on the immortalized cell lines utilized by the selected studies.

a/a	Reference	Complies with Essential Criteria (4)	Overall Score (Max 17)	Klimisch Category ^a^
1	Sharma, 2012 [58]	√	14	I ^b^
2	Tang, 2013 [60]	√	15	I
3	Yu, 2014 [61]	√	17	I
4	Huang, 2014 [63]	√	16	I
5	Cowger, 2015 [64]	√	13	II ^b^
6	Yu, 2015 [66]	√	15	I
7	Hasan, 2017 [68]	√	16	I
8	Ahmadpoor, 2018 [71]	√	16	I
9	Feng, 2018 [72]	√	14	I
10	Bordet, 2019 [73]	√	12	II ^b^
11	Yu, 2019 [74]	√	17	I
12	Gangwar, 2020 [75]	√	16	I
13	Sun, Zhao 2021 [76,77]	√	15	I
14	Ülküseven, 2023 [78]	√	17	I

^a^ Categories: I, reliable without restrictions; II, reliable with restrictions, and III, unreliable. ^b^ Studies not included in the meta-analysis.

**Table 5:** Overall and subgroup analysis of ICNPs treatment effects on cell viability.

Subgroup	Number of Experiments	Heterogeneity I^2^%	SMD (95% CI)	Overall *p*-Value ^a^
Organism/Species				0.2604
Human	115	75.0%	−2.791 [−3.383; −2.199]	
Murine	57	80.6%	−2.288 [−2.849; −1.225]	
Health Status				0.167
Normal Cells	44	78.9%	−2.037 [−2.849; −1.125]	
Cancer Cells	128	76.4%	−2.711 [−3.214; −2.208]	
ICNPs Ligand				<0.001
PEG-based	52	79.4%	−4.062 [−4.835; −3.289]	
Protein-based	52	80.6%	−2.280 [−3.092; −1.469]	
Other	36	36.7%	−0.572 [−0.914; −0.229]	
None	32	60.7%	−0.594 [−0.997; −0.191]	
Particle Size				0.533
<20 nm	53	73.7%	−2.761 [−3.577; 1.947]	
≥20 nm	119	78.5%	−2.456 [−2.965; −1.947]	
Concentration Range				<0.001
<10 μg/mL	29	72.4%	−0.651 [−1.209; −0.093]	
10–50 μg/mL	46	72.9%	−1.987 [−2.713; −1.261]	
50–100 μg/mL	23	53.4%	−1.185 [−1.589; −0.781]	
100–250 μg/mL	33	66.0%	−3.358 [−4.166; −2.551]	
>250 μg/mL	41	82.5%	−5.337 [−6.768; −3.906]	
Overall	172	77.1%	−2.531 [−2.959; −2.102]	<0.001

^a^ Intergroup heterogeneity of subgroups.

## Data Availability

The research data can be found in the figures and tables within this article and Supplementary Material.

## Supplementary Materials

The following supporting information can be downloaded at: https://www.mdpi.com/article/10.3390/nano14090734/s1, Table S1: Search strategy and query modifications for each database to identify relevant studies; Table S2: Exclusion reasons after full-text assessment; Table S3: Secondary outcomes and IC_50_ measurements, where available/applicable; Table S4: Criteria included in the reliability assessment tool ToxRTool for in vitro toxicity studies; Figure S1: Forest plots showing: (a) the effect of ICNP on cell viability in human and murine cell lines compared to control samples, and (b) the subgroup analysis based on tested organisms/ species; Figure S2: Forest plots showing: (a) the subgroup analysis based on the health status of the cell line, and (b) the subgroup analysis based on the ligands conjugated on the NPs; Figure S3: Forest plots showing: (a) the subgroup analysis based on particle size, and (b) the subgroup analysis based on concentration ranges; Table S5: Preferred Reporting Items for Systematic Reviews and Meta-Analyses (PRISMA) checklist.

## Abbreviations

ATP	Adenosine triphosphate
CDT	Chemodynamic therapy
CI	Confidence Interval
DOX	Doxorubicin
ES	Effect size
IC_50_	Half-maximum inhibitory concentration
ICNP	Iron carbide nanoparticle
IONP	Iron oxide nanoparticles
LDH	Lactate dehydrogenase
MHT	Magnetic Hyperthermia
MNP	Magnetic nanoparticles
MRI	Magnetic Resonance Imaging
MeSH	Medical Subject Headings
Ms	Magnetization Saturation
NIR	Near-infrared
NM	Nanomaterial
NP	Nanoparticle
OSF	Open Science Framework
PAT	Photoacoustic Tomography
PDT	Photodynamic therapy
PEG	Polyethylene glycol
PRISMA	Preferred reporting items for systematic review and meta-analyses
PTT	Photothermal therapy
ROS	Reactive Oxygen Species
SMD	Standardized Mean Difference
SR	Systematic Review
SRB	Sulforhodamine B
ZP	Zeta Potential
