# Supplementary material for: In Vitro Toxicological Insights from the Biomedical Applications of Iron Carbide Nanoparticles in Tumor Theranostics: A Systematic Review and Meta-Analysis
Source: Nanomaterials (Basel). 2024 Apr 23;14(9):734. doi: 10.3390/nano14090734 (PMC11085367; doi:10.3390/nano14090734)
Supplement: Supplementary file 1 [file nanomaterials-14-00734-s001.zip › nanomaterials-2959751-supplementary.pdf]

Supplementary Material

Table S1. Search strategy and query modifications for each database to identify relevant studies.

Resource	Search Query and Refinements	Post-query filter	Number of studies
Scopus	-Search within Article title, Abstract, Keywords:<br>"iron carbide" OR Fe3C OR Fe5C2 OR Fe2C OR Fe2.2C OR FexCy, AND<br>-Search within All fields:<br>cytotoxicity OR toxicity OR adverse OR "cell viability" OR "in vitro", AND<br>-Search within All fields:<br>biocompatibility OR biomedical OR nanomedicine OR theranostics OR "drug delivery" OR mri OR "contrast agent" OR hyperthermia	None	105
PubMed	(("iron carbide"[All Fields] OR "Fe3C"[All Fields] OR "Fe5C2"[All Fields] OR "Fe2C"[All Fields] OR "FexCy"[All Fields]) AND ("cytotox"[All Fields] OR "cytotoxic"[All Fields] OR "cytotoxical"[All Fields] OR "cytotoxically"[All Fields] OR "cytotoxicities"[All Fields] OR "cytotoxicity"[All Fields] OR "cytotoxics"[All Fields] OR "cytotoxities"[All Fields] OR "cytotoxity"[All Fields] OR ("toxic"[All Fields] OR "toxical"[All Fields] OR "toxically"[All Fields] OR "toxicant"[All Fields] OR "toxicant s"[All Fields] OR "toxicants"[All Fields] OR "toxicated"[All Fields] OR "toxication"[All Fields] OR "toxicities"[All Fields] OR "toxicity"[MeSH Subheading] OR "toxicity"[All Fields] OR "toxicity s"[All Fields] OR "toxics"[All Fields]) OR ("toxic"[All Fields] OR "toxical"[All Fields] OR "toxically"[All Fields] OR "toxicant"[All Fields] OR "toxicant s"[All Fields] OR "toxicants"[All Fields] OR "toxicated"[All Fields] OR "toxication"[All Fields] OR "toxicities"[All Fields] OR "toxicity"[MeSH Subheading] OR "toxicity"[All Fields] OR "toxicity s"[All Fields] OR "toxics"[All Fields]) OR ("adverse"[All Fields] OR "adversely"[All Fields] OR "adverses"[All Fields]) OR "cell viability"[All Fields] OR "in vitro"[All Fields] OR ("biocompatability"[All Fields] OR "biocompatibilities"[All Fields] OR "biocompatibility"[All Fields] OR "biocompatible"[All Fields] OR ("biomedical"[All Fields] OR "biomedically"[All Fields]) OR ("nanomedicinal"[All Fields] OR "nanomedicine"[MeSH Terms] OR "nanomedicine"[All Fields] OR "nanomedicines"[All Fields]) OR ("precision medicine"[MeSH Terms] OR ("precision"[All Fields] AND "medicine"[All Fields]) OR "precision medicine"[All Fields] OR "theranostic"[All Fields] OR "theranostics"[All Fields]) OR "drug delivery"[All Fields] OR ("magnetic resonance imaging"[MeSH Terms] OR ("magnetic"[All Fields] AND "resonance"[All Fields] AND "imaging"[All Fields]) OR "magnetic resonance imaging"[All Fields] OR "mri"[All Fields]) OR "contrast agent"[All Fields] OR ("hyperthermia"[MeSH Terms] OR "hyperthermia"[All Fields] OR "hyperthermias"[All Fields])))) AND (2010:2023[pdat])	None	41
Wiley Online Library	"("iron carbide" OR Fe3C OR Fe5C2 OR Fe2C OR Fe2.2C OR FexCy)" anywhere and "(cytotoxicity OR toxicity OR "cell viability" OR adverse OR "in vitro")" anywhere and "(biocompatibility OR biomedical OR nanomedicine OR mri OR "contrast agent" OR hyperthermia)" anywhere	-Type: Journals, -Subjects: Chemistry/ Chemical & Biochemical Engineering / Biomedical Engineering	168

Table S2. Exclusion reasons after full-text assessment

Study ID	Reason for exclusion	References (DOIs)
Davydov et al., 2014	Wrong population (pig kidney cells)	10.1039/c3tb21599g
Balfourier et al., 2023	Wrong outcome (evaluated the cytotoxicity only in vivo)	10.1002/smtd.202201061
Sun et al., 2023	Wrong exposure (use of ICNPs in microneedles	10.1002/adhm.202301474

Table S3. Secondary outcomes and IC50 measurements, where available/applicable. ZP, Zeta Potential (of the iron carbide nanomaterials); Ms, Magnetization saturation; NIR, Near infrared; IC_{50}, Half maximal inhibitory concentration.

Ref	Core/Shell structure	Ligand-Conjugate	ZP (mV)	Ms (emu/g)	NIR	IC_{50} (μg/mL)
1	$Fe_3C@C$	-	-	48	No	NA
2	$Fe_5C_2@Fe_3O_4$	DSPE-PEG-COOH	-	125.4	No	89.99
3	$Fe_5C_2@C$	Zher2:342	-	-	Yes	3.14
	$Fe_5C_2@C$	PEG	-	125	Yes	139.16
4	$Fe_5C_2@C$	ST	-	120	No	NA
5	Fe_5C_2	DSPE-PEG-COOH (PL)	-	-	No	NA
	Fe_5C_2	ZDS	-	-	No	NA
	Fe_5C_2	Casein	-	-	No	NA
6	$Fe_5C_2@C$	BSA-DOX	-5	-	Yes	150.27 (No NIR), 5.32 (NIR)
	$Fe_5C_2@C$	BSA	-13.5	112.74	Yes	NA (No NIR), 21.01 (NIR)
7	Fe_2C	PA	-30.63	88.2	No	NA
8	$Fe_5C_2@SiO_2$	-	-	74	No	184.783
9	$Fe_5C_2@MnO_2$	GOD	8.9	54	No	47.19
	$Fe_5C_2@MnO_2$	-	10	76	No	NA
10	$Fe_{2.2}C$	DOP-TEG-C6	-	160	No	38.47
	$Fe_{2.2}C$	DOP-TEG-COOH	-	-	No	NA
	$Fe_{2.2}C$	DOP-TEG-Zwitter	-	-	No	NA
11	$Fe_5C_2@Fe_3O_4$	DSPE-PEG	-	97	No	NA
	$Fe_5C_2@C$	DSPE-PEG	-	-	No	NA
12	Fe_3C	Pluronic acid F127	-12.3	78.2	No	NA
13	$Fe_2C@Fe_3O_4$	DSPE-PEG	-21.5	65.5	No	NA
14	$Fe_3C@C$	PAA	-	63.6	Yes	27.77

Abbreviations: DSPE-PEG, 1,2-Distearoyl-sn-glycero-3-phosphoethanolamin - Polyethylene Glycol; ST, Sodium Tartrate; PL, phospholipids; ZDS, Zwitterion Dopamine Sulfonate; BSA, Bovine Serum Albumin; DOX, Doxorubicin; PA, Protocatechuic Acid; GOD, Glucose Oxidase; DOP, Dopamine; TEG, Triethylene Glycol; PAA, Poly(acrylic acid); H, Human; M, Murine;

Table S4. Criteria included in the reliability assessment tool ToxRTool for *in vitro* toxicity studies applied to 14 publications resulting from the systematic review.

Criterion	(1*)	(2)	(3)	(4)	(5)	(6)	(7)	(8)	(9*)	(10*)	(11-12)	(13)	(14)	(15)	(16)	(17*)	(18)	Overall Score
Sharma	1	1	1	1	1	0	0	1	1	1	1	0	1	1	1	1	1	14
Tang	1	0	1	1	1	1	1	0	1	1	1	1	1	1	1	1	1	15
Yu (2014)	1	1	1	1	1	1	1	1	1	1	1	1	1	1	1	1	1	17
Huang	1	1	1	1	1	0	1	1	1	1	1	1	1	1	1	1	1	16
Cowger	1	1	1	1	1	0	0	0	1	1	1	1	1	1	0	1	1	13
Yu (2015)	1	1	1	1	1	1	1	1	1	1	1	1	1	0	1	1	0	15
Hasan	1	1	1	1	1	1	1	1	1	1	1	0	1	1	1	1	1	16
Ahmadpoor	1	1	1	1	1	0	1	1	1	1	1	1	1	1	1	1	1	16
Feng	1	1	1	1	1	1	1	1	1	1	0	1	1	0	1	1	0	14
Bordet	1	1	1	1	1	0	0	0	1	1	0	1	1	1	1	1	0	12
Yu (2019)	1	1	1	1	1	1	1	1	1	1	1	1	1	1	1	1	1	17
Gangwar	1	1	1	1	1	1	1	1	1	1	1	1	1	1	0	1	1	16
Sun & Zhao	1	1	1	1	1	1	1	1	1	1	1	1	1	1	1	1	1	17
Ülküseven	1	1	1	1	1	1	1	1	1	1	1	1	1	1	1	1	1	17

(1)Test substance identification; (2) substance purity statement; (3) source/origin information of the substance; (4) information on physicochemical properties of the test item given; (5) cell culture description; (6) the source/origin of cell culture; (7) necessary information on cell culture properties, conditions of cultivation and maintenance; (8) the method of nanomaterial administration; (9) doses or concentration statement; (10) frequency and duration of exposure and time-points of observations statement; (11-12) has negative controls and/or positive controls; (13) the number of replicates is provided; (14) are the study endpoints and their methods of determination clearly described?; (15) is the description of the study results for all endpoints investigated transparent and complete?; (16) are the statistical methods for data analysis given and applied in a transparent manner?; (17) is the study design chosen appropriate for obtaining the substance-specific data aimed at?; (18) are the quantitative study results reliable?

Compulsory questions are indicated with an asterisk (*)

(a) **(b)**

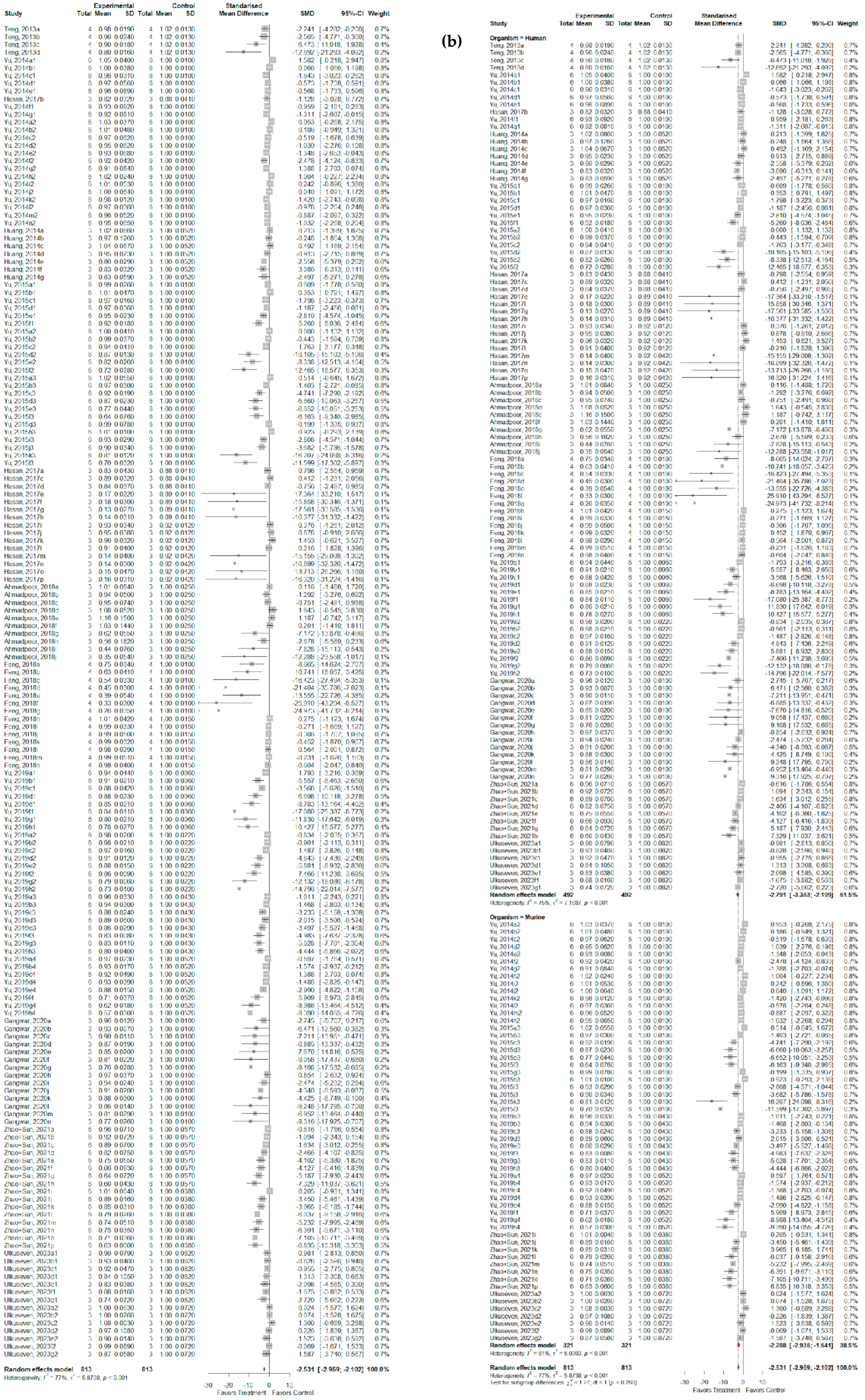

Figure S1. Forest plots showing: (a) the effect of ICNP on cell viability in human and murine cell lines compared to control samples, and (b) the subgroup analysis based on tested organisms/species.

(a)

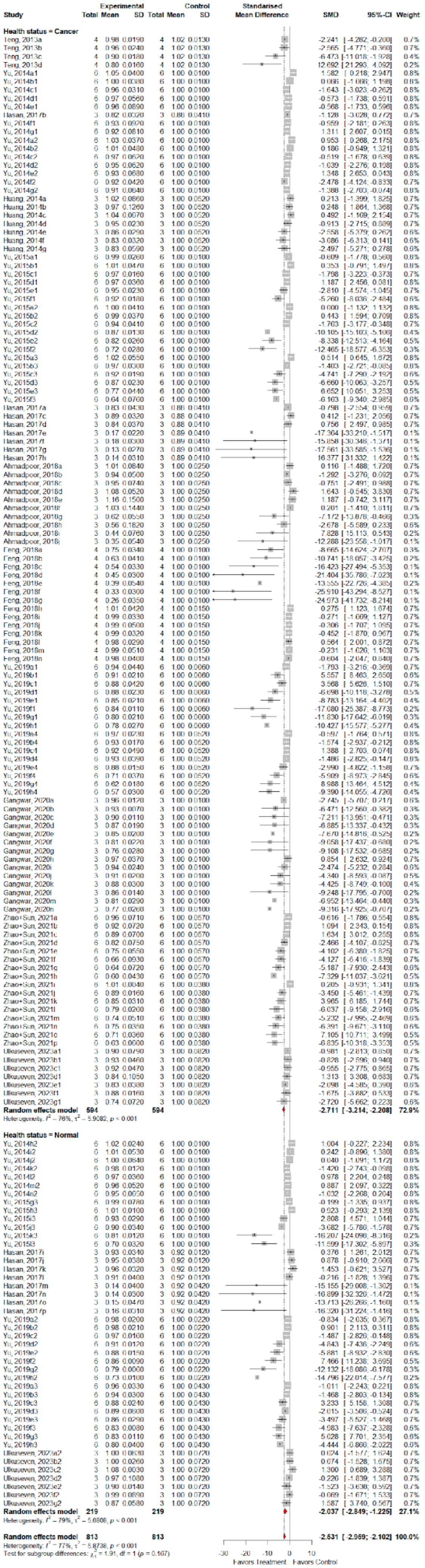

(b)

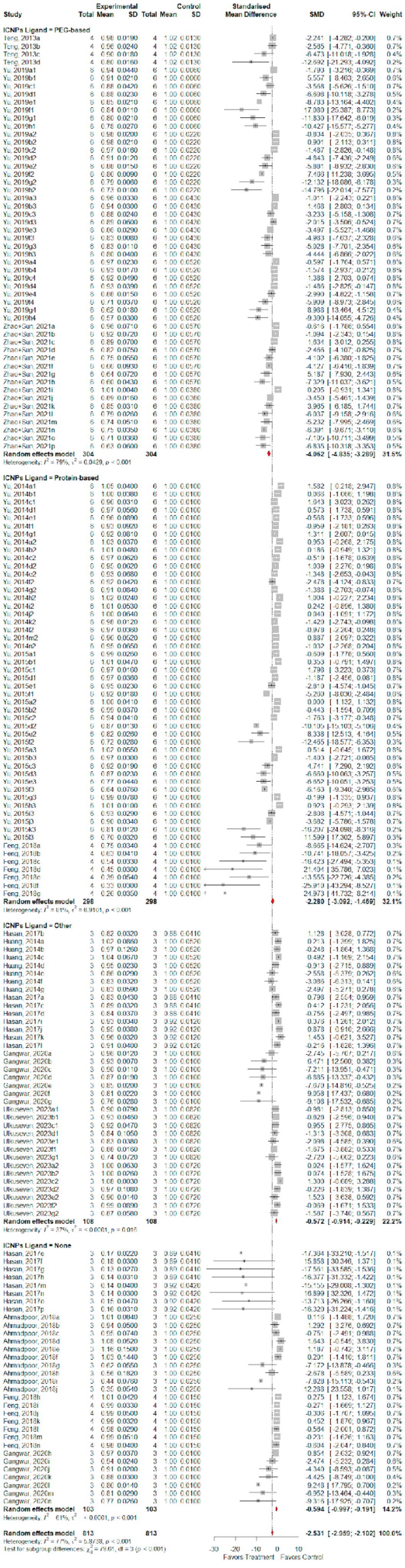

Figure S2. Forest plots showing: (a) the subgroup analysis based on the health status of the cell line, and (b) the subgroup analysis based on the ligands conjugated on the NPs.

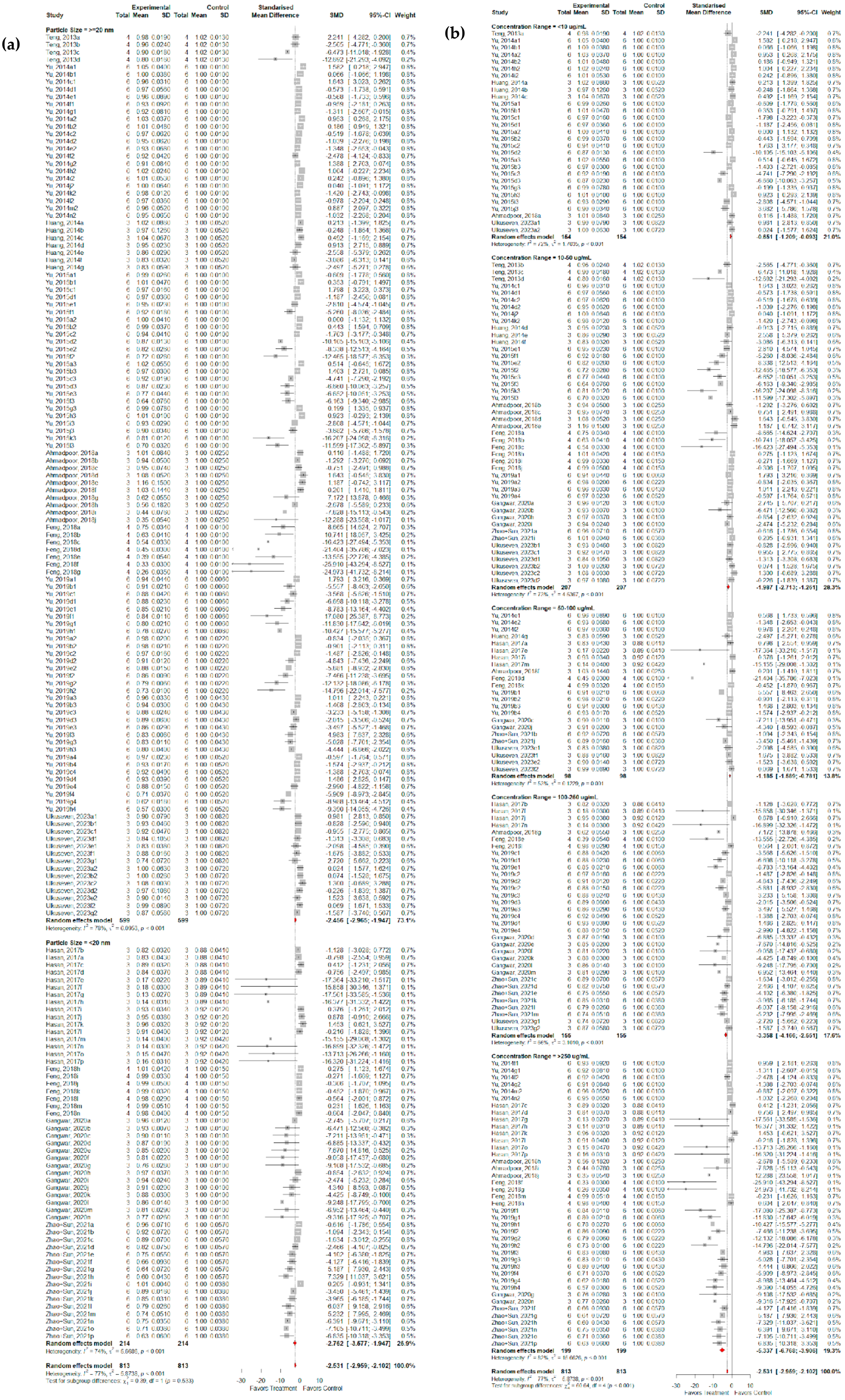

Figure S3. Forest plots showing: (a) the subgroup analysis based on particle size, and (b) the subgroup analysis based on concentration ranges.

Table S5. Preferred Reporting Items for Systematic Reviews and Meta-Analyses (PRISMA) checklist.

Section and Topic	Item #	Checklist item	Location where item is reported*
**TITLE**			
Title	1	Identify the report as a systematic review.	Title (Page 1)
**ABSTRACT**			
Abstract	2	See the PRISMA 2020 for Abstracts checklist.	Abstract (Page 1)
**INTRODUCTION**			
Rationale	3	Describe the rationale for the review in the context of existing knowledge.	Introduction (Page 1)
Objectives	4	Provide an explicit statement of the objective(s) or question(s) the review addresses.	Introduction (Page 1), Study Design and Protocol (Page 3)
**METHODS**			
Eligibility criteria	5	Specify the inclusion and exclusion criteria for the review and how studies were grouped for the syntheses.	Eligibility Criteria (Page 3)
Information sources	6	Specify all databases, registers, websites, organisations, reference lists and other sources searched or consulted to identify studies. Specify the date when each source was last searched or consulted.	Search Strategy (Page 4)
Search strategy	7	Present the full search strategies for all databases, registers and websites, including any filters and limits used.	Suppl. Material (Table S1)
Selection process	8	Specify the methods used to decide whether a study met the inclusion criteria of the review, including how many reviewers screened each record and each report retrieved, whether they worked independently, and if applicable, details of automation tools used in the process.	Screening and Selection Process (Page 5)
Data collection process	9	Specify the methods used to collect data from reports, including how many reviewers collected data from each report, whether they worked independently, any processes for obtaining or confirming data from study investigators, and if applicable, details of automation tools used in the process.	Screening and Selection Process, Data Extraction (Page 5)
Data items	10a	List and define all outcomes for which data were sought. Specify whether all results that were compatible with each outcome domain in each study were sought (e.g. for all measures, time points, analyses), and if not, the methods used to decide which results to collect.	Data Extraction (Page 5)
	10b	List and define all other variables for which data were sought (e.g. participant and intervention characteristics, funding sources). Describe any assumptions made about any missing or unclear information.	Data Extraction (Page 6)
Study risk of bias assessment	11	Specify the methods used to assess risk of bias in the included studies, including details of the tool(s) used, how many reviewers assessed each study and whether they worked independently, and if applicable, details of automation tools used in the process.	Critical Appraisal (Page 6)
Effect measures	12	Specify for each outcome the effect measure(s) (e.g. risk ratio, mean difference) used in the synthesis or presentation of results.	Statistical Analysis (Page 7)
Synthesis methods	13a	Describe the processes used to decide which studies were eligible for each synthesis (e.g. tabulating the study intervention characteristics and comparing against the planned groups for each synthesis (item #5)).	Eligibility Criteria (Page 3), Meta-Analysis (Page 13)
	13b	Describe any methods required to prepare the data for presentation or synthesis, such as handling of missing summary statistics, or data conversions.	Data Extraction (Page 5)
	13c	Describe any methods used to tabulate or visually display results of individual studies and syntheses.	Statistical Analysis

Section and Topic	Item #	Checklist item	Location where item is reported*
			(Page 7)
	13d	Describe any methods used to synthesize results and provide a rationale for the choice(s). If meta-analysis was performed, describe the model(s), method(s) to identify the presence and extent of statistical heterogeneity, and software package(s) used.	Statistical Analysis (Page 7)
	13e	Describe any methods used to explore possible causes of heterogeneity among study results (e.g. subgroup analysis, meta-regression).	Statistical Analysis (Page 7)
	13f	Describe any sensitivity analyses conducted to assess robustness of the synthesized results.	N.A.
Reporting bias assessment	14	Describe any methods used to assess risk of bias due to missing results in a synthesis (arising from reporting biases).	Statistical Analysis (Page 7)
Certainty assessment	15	Describe any methods used to assess certainty (or confidence) in the body of evidence for an outcome.	N.A.
**RESULTS**			
Study selection	16a	Describe the results of the search and selection process, from the number of records identified in the search to the number of studies included in the review, ideally using a flow diagram.	Literature Search (Page 7, Figure 3)
	16b	Cite studies that might appear to meet the inclusion criteria, but which were excluded, and explain why they were excluded.	Suppl. Material (Table S2)
Study characteristics	17	Cite each included study and present its characteristics.	Characteristics of the included studies (Page 8, Tables 1&2)
Risk of bias in studies	18	Present assessments of risk of bias for each included study.	Quality Assessment (Page 12), Suppl. Material (Table S4)
Results of individual studies	19	For all outcomes, present, for each study: (a) summary statistics for each group (where appropriate) and (b) an effect estimate and its precision (e.g. confidence/credible interval), ideally using structured tables or plots.	Suppl. Material (Fig. S1(a))
Results of syntheses	20a	For each synthesis, briefly summarise the characteristics and risk of bias among contributing studies.	Suppl. Material (Fig. S1(b)-3)
	20b	Present results of all statistical syntheses conducted. If meta-analysis was done, present for each the summary estimate and its precision (e.g. confidence/credible interval) and measures of statistical heterogeneity. If comparing groups, describe the direction of the effect.	Meta-Analysis (Page 13)
	20c	Present results of all investigations of possible causes of heterogeneity among study results.	Meta-Analysis (Table 5)
	20d	Present results of all sensitivity analyses conducted to assess the robustness of the synthesized results.	N.A.
Reporting biases	21	Present assessments of risk of bias due to missing results (arising from reporting biases) for each synthesis assessed.	Meta-Analysis (Figure 5)
Certainty of evidence	22	Present assessments of certainty (or confidence) in the body of evidence for each outcome assessed.	N.A.
**DISCUSSION**			
Discussion	23a	Provide a general interpretation of the results in the context of other evidence.	Discussion (Page 15), Meta-Analysis Outcomes (Page 16)

Section and Topic	Item #	Checklist item	Location where item is reported*
	23b	Discuss any limitations of the evidence included in the review.	Strengths and Limitations (Page 16)
	23c	Discuss any limitations of the review processes used.	Strengths and Limitations (Page 16)
	23d	Discuss implications of the results for practice, policy, and future research.	Conclusion (Page 17)
**OTHER INFORMATION**			
Registration and protocol	24a	Provide registration information for the review, including register name and registration number, or state that the review was not registered.	Study design and Protocol (Page 3)
	24b	Indicate where the review protocol can be accessed, or state that a protocol was not prepared.	Study design and Protocol (Page 3)
	24c	Describe and explain any amendments to information provided at registration or in the protocol.	Quality Assessment (Page 12)
Support	25	Describe sources of financial or non-financial support for the review, and the role of the funders or sponsors in the review.	Funding (Page 17)
Competing interests	26	Declare any competing interests of review authors.	Conflicts of interest (Page 17)
Availability of data, code and other materials	27	Report which of the following are publicly available and where they can be found: template data collection forms; data extracted from included studies; data used for all analyses; analytic code; any other materials used in the review.	Data Availability (Page 17)

*N.A. Not Applicable.
